# Advances in spatial transcriptomic data analysis

**DOI:** 10.1101/gr.275224.121

**Published:** 2021-10

**Authors:** Ruben Dries, Jiaji Chen, Natalie del Rossi, Mohammed Muzamil Khan, Adriana Sistig, Guo-Cheng Yuan

**Affiliations:** 1 Department of Medicine, Boston University School of Medicine, Boston, Massachusetts 02118, USA;; 2 Bioinformatics Graduate Program, Boston University, Boston, Massachusetts 02215, USA;; 3 Section of Computational Biomedicine, Boston University School of Medicine, Boston, Massachusetts 02118, USA;; 4 Department of Genetics and Genomic Sciences, Charles Bronfman Institute for Personalized Medicine, Icahn School of Medicine at Mount Sinai, New York, New York 10029, USA;; 5 Precision Immunology Institute, Icahn School of Medicine at Mount Sinai, New York, New York 10029, USA

## Abstract

Spatial transcriptomics is a rapidly growing field that promises to comprehensively characterize tissue organization and architecture at the single-cell or subcellular resolution. Such information provides a solid foundation for mechanistic understanding of many biological processes in both health and disease that cannot be obtained by using traditional technologies. The development of computational methods plays important roles in extracting biological signals from raw data. Various approaches have been developed to overcome technology-specific limitations such as spatial resolution, gene coverage, sensitivity, and technical biases. Downstream analysis tools formulate spatial organization and cell–cell communications as quantifiable properties, and provide algorithms to derive such properties. Integrative pipelines further assemble multiple tools in one package, allowing biologists to conveniently analyze data from beginning to end. In this review, we summarize the state of the art of spatial transcriptomic data analysis methods and pipelines, and discuss how they operate on different technological platforms.

Multicellular organisms consist of tissues and organs, each specializing in a subset of biological processes and performed by the coordinated activities of many cells. Although all normal cells share the same genome, their gene expression patterns and morphology can be drastically different. This variation is caused not only by internal gene regulatory circuitry differences but also by signaling from the external tissue environment. Whereas decades of genome-wide studies have accumulated large amounts of information about cell type–specific gene regulatory circuitries, our understanding of the external cell–tissue environment interactions remains limited.

Recent years have witnessed an explosion of technological advances that collectively enable system-level characterization of cellular heterogeneity and spatial organization of tissues/organs. Perhaps most notably is the rapid development of single-cell RNA-seq technology (scRNA-seq) applications that made it possible to profile and compare the gene expression patterns of a large number of individual cells within a tissue/organ (Svensson et al. 2018b). Together with the development of a rich set of computational methods for data analysis (for review, see Yuan 2019; Hie et al. 2020), the scRNA-seq field has fulfilled a key role in the discovery of novel cell types and laid the foundation for the creation of comprehensive cell atlases in different species (Han et al. 2018, 2020; Sebé-Pedrós et al. 2018; Spanjaard et al. 2018; Tabula Muris Consortium et al. 2018; Cao et al. 2019; Packer et al. 2019; Pijuan-Sala et al. 2019). However, a key step in the experimental process is the creation of a single-cell suspension through mechanical and enzymatic dissociation steps, which inherently destroys the original tissue architecture.

As such, reconstructing the structure of a tissue from its cellular components alone is extremely difficult, if not impossible. Just like putting together a complex jigsaw puzzle from individual pieces, the precise position and organization of the cells matter. The tissue environment plays a critical role during development in which, for example, it defines asymmetric cell fate decisions and instructs cell movement. Positional information continues to be crucial at the adult stage to exert tissue-specific functions, to maintain tissue homeostasis, and to respond to external cues or perturbations. Notably, in diseases such as cancer, the normal tissue environment can be reprogrammed and manipulated to promote malignant cell expansion, which is normally suppressed (Whiteside 2008), whereas deep understanding of the tumor immune environment is essential for developing effective immunotherapeutic approaches (Binnewies et al. 2018).

During the past few years, various technologies have been developed for transcriptomic profiling while preserving spatial information. Collectively, these technologies have been named as the method of the year of 2020 by *Nature Methods* (Marx 2021) to recognize their importance and are expected to rapidly transform biological research in the coming years. Currently, there exist three major approaches that are engaged to spatially explore large pieces of tissue and aim to perform this at single-cell resolution and on a genome-wide scale. First, sequential fluorescent in situ hybridization (FISH)–based methods use a targeted approach, which is based on predesigned probes. By introducing clever barcoding strategies combined with sequential hybridization and imaging, they can identify the exact position of tens to thousands of individual transcripts within a fixed tissue specimen (Lubeck et al. 2014; Chen et al. 2015; Shah et al. 2016; Codeluppi et al. 2018; Moffitt et al. 2018; Eng et al. 2019; Kishi et al. 2019; Goh et al. 2020). Second, spatial labeling technologies use ingenious ways to link all transcripts within a spatial unit with known coordinates. This in situ capturing step is subsequently followed by an unbiased standard sequencing approach (Ståhl et al. 2016; Rodriques et al. 2019; Vickovic et al. 2019; Liu et al. 2020; Merritt et al. 2020; Chen et al. 2021; Cho et al. 2021; Stickels et al. 2021). Third, select genes can be targeted for in situ sequencing (ISS), with the synthesized cDNA products labeled by fluorescent nucleotides and detected by imaging (Lee et al. 2014; Wang et al. 2018; Qian et al. 2020; Hu et al. 2020b; Alon et al. 2021; Fu et al. 2021). For more detailed information about the different spatial units and the capturing and linking strategies, the reader is referred to spatial technology reviews (Asp et al. 2020; Liao et al. 2021). Here we use four data sets to illustrate the outcome or methodology of different spatial transcriptomic analysis steps: (1) a genome-wide spatial transcriptomics data set generated from multiple slices of a breast tumor biospecimen (Andersson et al. 2020b), (2) a subcellular spatial data set from a whole-mouse coronal brain slice with approximately 500 genes across about 78,000 generated by VIZGEN with MERFISH technology (https://info.vizgen.com/mouse-brain-data), (3) a genome-wide spatial data set from the human heart created by the Visium platform from 10x Genomics (https://support.10xgenomics.com/spatial-gene-expression/datasets/1.1.0/V1_Human_Heart), and (4) another subcellular spatial data set covering 10,000 genes from hundreds of cells within the mouse somatosensory cortex and generated by the seqFISH+ technology (Fig. 1; Eng et al. 2019).

**Figure 1.**
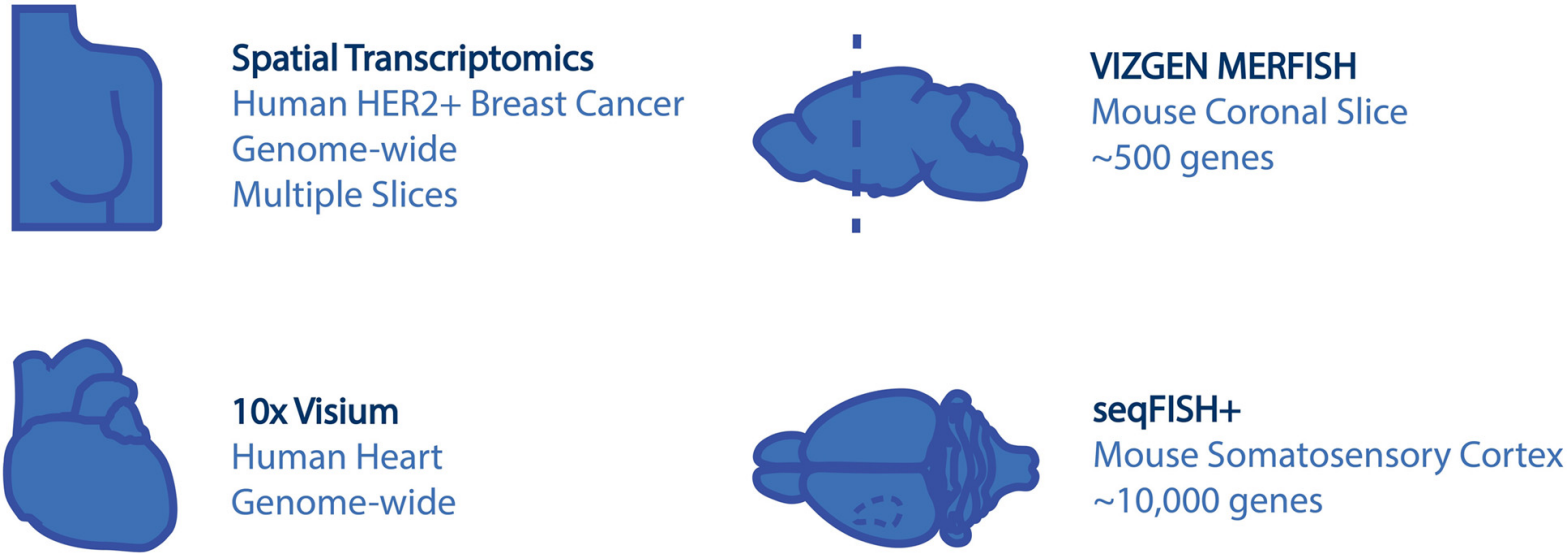
Data sets used in this Perspective.

Obtaining a gene expression matrix and corresponding spatial coordinates from a raw ST data set is generally not a trivial process and consists of a number of preprocessing steps. These steps are typically technology or platform dependent, but there are a few recurring preprocessing steps that are inherent to some or all technologies, such as image registration, stitching, and cell segmentation for data based on imaging (Fig. 2).

**Figure 2.**
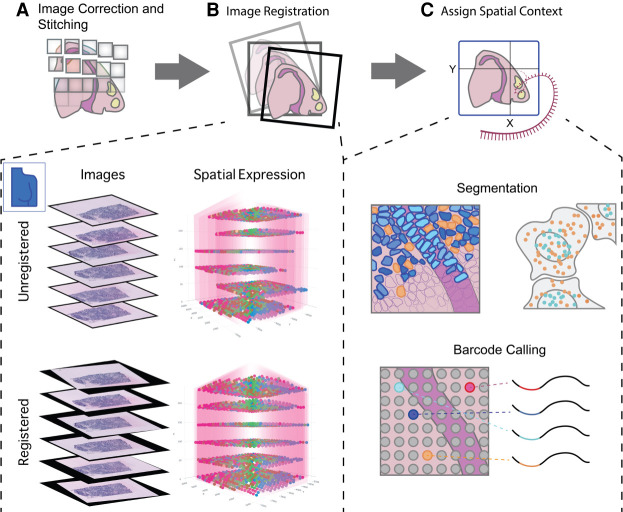
Preprocessing of raw spatial transcriptomic data. (*A*) For spatial transcriptomics data paired with images, processing begins with correction and stitching of multiple captures or fields of views (FOVs) to form a clear composite image. (*B*) Images from multiple stacked sections of the same tissue can be registered and the resulting spatial transformations mapped back to the transcriptomic data in order to create an aligned 3D gene expression data set. This is illustrated with the breast cancer spatial transcriptomics data set from Andersson et al. (2020b). (*C*) Several methods exist to provide expression data with spatial context. For technologies such as FISH and ISS that do not have clearly defined read spots or boundaries, cell segmentation (*upper* panel) is required in order to assign reads to individual cells. In situ capture or array-based methods, on the other hand (*lower* panel), assign reads to read spots based on a spatial barcode unique to each spatial unit (e.g., spot).

For imaging-based ST data, such as the FISH and ISS technologies, the most frequent image processing steps are image correction, stitching, registration, segmentation, followed by locating and decoding individual spots that usually correspond to a single transcript. Initial corrections to the obtained images are almost always needed to adjust for technological artifacts and are often dependent on the experimental assay. The main goal here is to increase the signal-to-noise ratio and create normalized intensities for further downstream steps. Multiple overlapping fields of view, or tiles, are needed when the tissue to be analyzed is too big in size, and they need to be stitched back together (Fig. 2A). Similarly, images that consist of multiple z-stacks can be misaligned because of technical or experimental procedures. For example, this occurs when multiple hybridization rounds are sequentially imaged or when creating a 3D data set by using adjacent 2D slices. The process to correct for this misalignment is often referred to as image registration and can be performed using a variety of different transformation algorithms and strategies (Fig. 2B; Borovec et al. 2020). As a demonstrating example, registration transformation is applied to a ST breast cancer data set (Andersson et al. 2020b), in which six sections were taken serially with a distance of 48 microns from each other. Applying registration transformation on all sections results in a clear visual improvement of the vertical alignment of the spatial expression data (Fig. 2B).

Both FISH- and ISS-based techniques provide single-cell or even subcellular resolution; however, this depends on proper identification of cell morphology. Tracing the cell boundaries—and other cellular structures such as the nucleus—is often referred to as cell segmentation (Fig. 2C). Although cell segmentation may appear rather simple to human eyes, it has been proven hard to automate. Segmentation difficulties are further aggravated by factors such as cell density (e.g., solid tumors) or complex cell shapes (e.g., neurons). A large number of methods have already been developed with gradual improvements in the accuracy and quality (for reviews, see Dimopoulos et al. 2014; Vicar et al. 2019). More recently, with the surge in deep learning frameworks and applications, there have been some considerable improvements in the creation of generalizable cell segmentation and image registration tools (Schmidt et al. 2018; Berg et al. 2019; Falk et al. 2019; Perkel 2019; Greenwald et al. 2021; Stringer et al. 2021). Finally, each spot needs to be identified and uniquely assigned to a gene. This decoding strategy is typically intertwined with the technological setup and design, but there are efforts to make this more generalizable and available to the broad community (Fig. 2C; Perkel 2019).

On the other hand, there are data that do not necessarily require imaging but rather operate through capturing transcripts within a defined spatial unit and linking them with a known coordinate system before the sequencing step. As such, these approaches are typically less—or not—dependent on the raw image processing steps described above. However additional steps are needed after sequencing to map the transcript back to their spatial coordinates. When accompanying tissue images are available, they may be overlaid with the spatial coordinate system. The readers are referred to the original protocols for more information about the data preprocessing procedures.

Regardless of technological differences, a common goal in ST analysis is to connect and integrate information from both gene expression and cellular or transcript locations. This is crucial for extracting useful biological information, allowing linking with cell morphology and generating new hypotheses (Fig. 3). In the following sections we will review the state-of-the-art computational methods and tools for these analyses. A curated list with additional details for all discussed methods is also provided at GitHub (https://github.com/drieslab/awesome-spatial-data-analysis).

**Figure 3.**
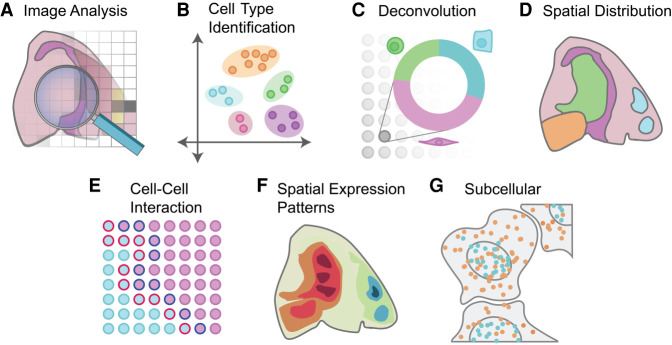
Overview of spatial transcriptomics analysis methods. A variety of analyses can be performed on spatial transcriptomics data. (*A*) Analysis can be performed on the image itself, ranging from early tasks such as cell segmentation to support of subcellular analysis through cell shape and size classification. (*B*) Cell types can be identified through clustering and annotation. Additional integration with external scRNA-seq data or deconvolution of spatial units that cover multiple cells (*C*) can be performed to fine-tune cell type mapping. (*D*) The spatial distribution of cell types and the underlying cell-to-cell communication (*E*) can be computed. (*F*) Spatial expression patterns are identified and visualized based on information of gene expression and spatial coordinates. (*G*) Data at subcellular resolution can be used to identify spatial and temporal dynamics of transcripts within a single cell.

## Identification of cell types from ST data

**Figure 4.**
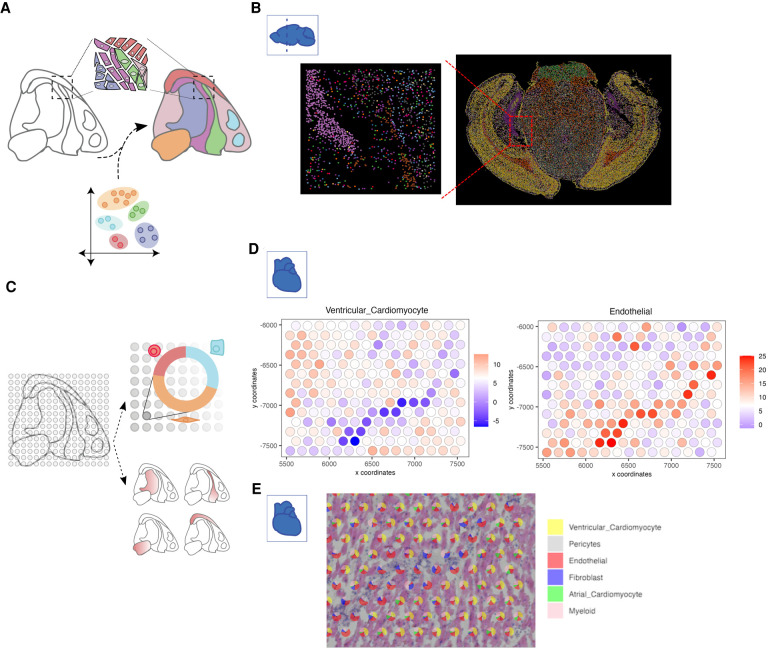
Strategies for cell type identification with spatial transcriptomic data. (*A*) Spatial transcriptomics data at single-cell resolution can be directly used to identify cell types in an analogous manner to scRNA-seq. In addition, external scRNA-seq from matching tissue can also be integrated to increase the number of available features and aid in the identification of detected cell types. (*B*) An example of cell type annotation is shown on the MERFISH mouse coronal brain slice data set. Each single dot represents a single cell, and colors indicate different cell types identified through clustering. A zoomed-in subset shows the spatial cell type composition at a higher resolution. (*C*) Cell types in non-single-cell spatial transcriptomic data are identified through deconvolution approaches that make use of external information or through gene enrichment strategies using sets of known marker genes or scRNA-seq information. (*D*) Enrichment scores for two cell types within the human heart 10x Genomics Visium data set are overlaid on top of the spots within a region of interest. (*E*) Pie charts depict the proportion of identified cell types within each selected spot used in *D*.

Cell type identification and localization is probably the most basic task for ST data analysis. If the data has single-cell resolution, such as in multiplexed FISH approaches (Lubeck et al. 2014; Chen et al. 2015; Shah et al. 2016; Codeluppi et al. 2018; Moffitt et al. 2018; Eng et al. 2019; Kishi et al. 2019; Goh et al. 2020), unsupervised clustering combined with manual or automatic annotation is a common approach to identify cell types in an unbiased manner (Fig. 4A). Because the spatial information is not needed for cell type identification, the task is highly similar to scRNA-seq analysis, for which numerous methods have been developed (for a benchmark study, see Abdelaal et al. 2019). For example, community-based methods such as Louvain (Blondel et al. 2008) and Leiden clustering (Traag et al. 2019) are popular choices for cell type identification, in which the clustering results are used as initial guide followed by often tedious manual biological annotations or through automated workflows as recently discussed by Pasquini et al. (2021). To show this approach, we used the MERFISH coronal slice data set and applied Leiden clustering, resulting in a total of 19 distinct clusters. These clusters are then annotated and mapped back to the spatial coordinates (Fig. 4B).

Although it is possible to use a sequential FISH approach to generate transcriptome-scale profiles (Eng et al. 2019; Xia et al. 2019), owing to the additional technical challenge, the common practice is to target a limited number of genes (typically only a few hundreds), which are often selected based on prior biological knowledge. As a consequence, the data are insufficient to discover unknown cell types in an unbiased manner but allow the biologists to annotate cell types whose gene signature is already known, often through external scRNA-seq analysis. Although the simplest approach is to identify the cell type whose gene signature has the highest correlation, a drawback is that it does not distinguish cell type marker genes from the transcriptome-wide background. Numerous computational approaches have been developed to optimize accuracy. For example, one approach is to build a support vector machine classifier based on the scRNA-seq data but only use information from the subset of genes that is also profiled in seqFISH (Zhu et al. 2018). A likelihood ratio test can also be used (Vickovic et al. 2019). Importantly, cross-platform normalization is needed to calibrate signals detected from different technologies. More generally, platform-specific technical variations can be estimated and reduced (Butler et al. 2018; Haghverdi et al. 2018; Barkas et al. 2019; Hie et al. 2019; Korsunsky et al. 2019; Stuart et al. 2019; Welch et al. 2019). Furthermore, Bayesian models have been developed to incorporate the impact of cell segmentation uncertainty on cell type annotations (Qian et al. 2020). Apart from cell type annotation, methods have also been developed to impute transcriptome-wide gene expression levels by integration with scRNA-seq data (Lopez et al. 2019; Lohoff et al. 2020).

Commercially available, array-based ST technologies (such as 10x Genomics Visium and NanoString GeoMx) typically do not have single-cell resolution. Because the variation of gene expression profiles may be associated with changes of cell type composition rather than new cell types, it is not appropriate to apply a clustering algorithm directly to such data and interpret the resulting clusters as cell types. Furthermore, it is possible to estimate cell type composition only if the underlying gene expression signatures are known. There are two general approaches for estimating cell type composition (Fig. 4C). The first approach is to evaluate the enrichment of cell type–specific markers among the expressed genes at each spot (Moncada et al. 2020; Dries et al. 2021). This approach is fast and can be performed one cell type at a time. However, the results are qualitative, indicating the presence or absence of a cell type. The second approach, deconvolution, aims to quantitatively estimate the proportion of different cell types at each location. Many deconvolution methods have been developed and benchmarked for RNA-seq data analysis (Avila Cobos et al. 2020). In principle, these tools can also be applied to ST analysis. On the other hand, ST data have certain distinct properties; for example, the number of cells associated with each location is often small. Therefore, it is often more accurate to use methods that are tailored for ST analysis (Andersson et al. 2020a; Biancalani et al. 2020; Kleshchevnikov et al. 2020; Cable et al. 2021; Dong and Yuan 2021; Elosua-Bayes et al. 2021; Lopez et al. 2021; Song and Su 2021). Among these methods, RCTD uses a linear regression model for gene counts, which further incorporates a random-effect term for platform-specific variations (Cable et al. 2021). The gene expression levels are modeled by a Poisson distribution. A similar approach is used in *stereoscope* (Andersson et al. 2020a). Cell2location uses a similar approach but models gene expression using the negative binomial distribution (Kleshchevnikov et al. 2020). It can also model platform- and location-specific effects. SpatialDWLS uses a two-step procedure to reduce noise (Dong and Yuan 2021). The first step identifies cell types that are likely to be present, by using an enrichment analysis as described above, and then the second step quantifies the relative proportion of each cell type by using a dampened weighted least-square procedure previously developed for RNA-seq data deconvolution (Tsoucas et al. 2019). SPOTlight uses a seeded nonnegative matrix factorization (NMF) regression, initialized using cell type marker genes and nonnegative least squares (NNLS) for subsequent deconvolution (Elosua-Bayes et al. 2021). DSTG uses a graph-based convolutional network approach (Song and Su 2021). DestVI uses a variational inference approach for deconvolution (Lopez et al. 2021). As an illustrating example, we use the Visium heart data set and matching scRNA-seq data (Litviňuková et al. 2020) to perform both cell type enrichment (Fig. 4D) and spatial deconvolution (Fig. 4E). Visualizing cell type enrichment is performed for each set of signature genes, whereas deconvolution results in a quantitative assessment of cell type composition for each spot.

A complementary approach to study cell type localization is to use scRNA-seq data as the starting point and then reconstruct spatial information based on similarities with spatial expression profiles. Before the explosion of ST technologies, it was possible to obtain spatial information only for a handful of landmark genes using traditional methods. Using such limited information, two groups were able to reconstruct transcriptome-wide spatial patterns using clever computational modeling (Achim et al. 2015; Satija et al. 2015). Around the same time, tomo-seq and Geo-seq technologies were developed to reconstruct 3D patterns from gene expression profiles obtained from 2D slices (Junker et al. 2014; Peng et al. 2016). A key missing link is that the spatial information is not directly measured from data; therefore, the patterns inferred from these analyses remain speculative. With the rapid development of ST technologies in the past few years, it is now possible to measure spatial information directly and further integrate with scRNA-seq data for additional refinement. Therefore, newer approaches integrate scRNA-seq and ST data in a more balanced manner. For example, a platform-agnostic, mutual nearest neighbor (MNN) approach has been used to align these data types, which results in cell locations mapping (Haghverdi et al. 2018; Hie et al. 2019; Stuart et al. 2019). DEEPsc uses an artificial neural network to predict spatial locations (Maseda et al. 2021). GLUER combines joint NMF, MNN algorithm, and deep neural network to align data (Peng et al. 2021). Tangram aligns scRNA-seq and ST data sets while optimizing the spatial correlation between each gene in the scRNA-seq data and in the spatial data (Biancalani et al. 2020). A similar idea is also implemented in NovaSparc (Nitzan et al. 2019) and D-CE (Zhao et al. 2021b). Of note, the alignment can be either probabilistic or deterministic. With the additional assumption that the total number of cells is known (which can be extracted from the H&E staining information), the deterministic mode of Tangram alignment also serves as a deconvolution method.

## Characterizing spatial patterns of transcriptomic profiles

**Figure 5.**
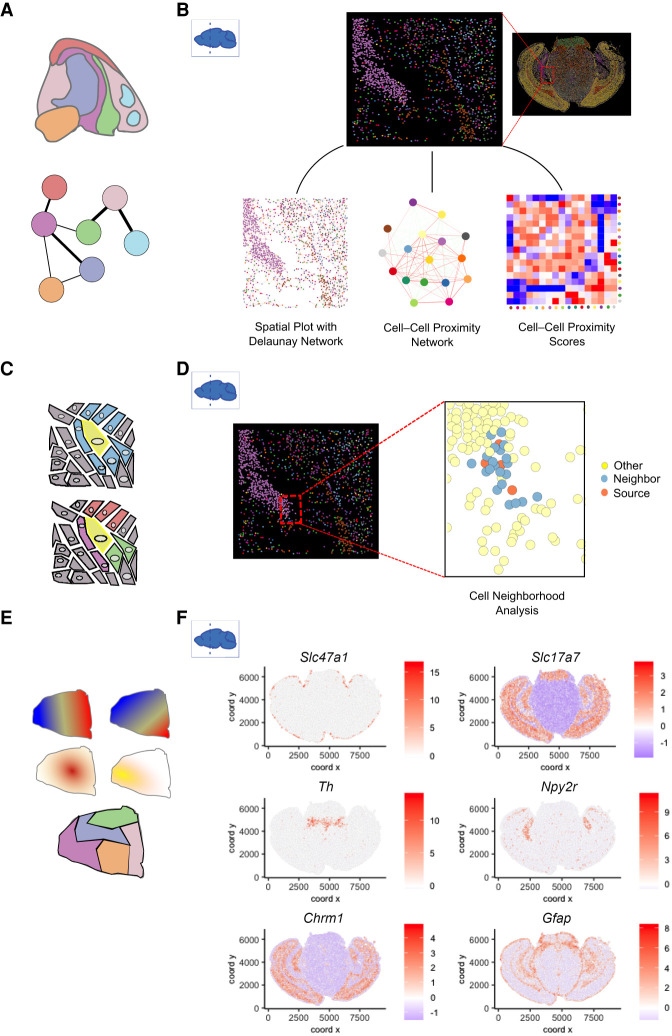
Spatial pattern analyses. (*A*) Spatial distribution analysis of neighboring cell types. Network represents the likelihood of two cell types being found in close physical proximity to each other. (*B*) A subset of cells from the MERFISH mouse coronal brain slice data set shows the spatial network connectivity and cellular proximities between different cell types. (*C*) At the single-cell level, cellular niches can be identified based on a target cell (yellow) and its direct neighboring cells (blue). The composition and position of the neighboring cell types create a niche for the target cell (*bottom*). (*D*) Source and neighboring cells are depicted within a small subset of the MERFISH mouse coronal brain slice data set. (*E*) Patterns based on spatial gene expression information are based on single or multiple genes and are continuous (*top*) or discrete (*bottom*). (*F*) Individual genes with unique spatial coherent expression patterns in the MERFISH mouse brain coronal data set are shown on the *right*.

The key contribution of ST analysis is to characterize not just the cell types but also how they are spatially organized. This is fundamentally important for studying the impact of tissue architecture and cell–cell interactions (Fig. 5A,C,E). To study the spatial patterns associated with gene expression and cell states, pairwise enrichment analysis can be used to identify cell type pairs that are likely to be next to each other (Schapiro et al. 2017; Dries et al. 2021). Cell neighborhood motif analysis identifies recurrent patterns of multiple cell type neighborhoods (Goltsev et al. 2018). An alternative approach to identify enriched patterns is to use topic models (Chen et al. 2020). Furthermore, the continuity of cell states can be incorporated into a hidden Markov random field (HMRF) model to identify coherent spatial domains (Zhu et al. 2018). This approach has been extended in more recent studies (Chidester et al. 2021; Zhao et al. 2021a). BayesSpace (Zhao et al. 2021a) uses a Bayesian formulation of HMRF, and the model parameters are estimated by a Markov chain Monte Carlo (MCMC) algorithm, whereas SPICEMIX (Chidester et al. 2021) combines HMRF with NMF. staNMF combines NMF with a stability criterion study to identify spatial patterns (Wu et al. 2016). To illustrate how spatial network patterns and cellular neighborhoods are studied, we used the MERFISH coronal slice data and created a cell–cell proximity network based on the physical coordinates of each cell that are connected through Delaunay triangulation. The cell–cell proximity network along with the heatmap shows the closeness and connectivity between different cell types and informs users about the spatial topology of the studied tissue (Fig. 5B). A detailed exploration of individual niches is shown in Figure 5D. Here, specific cells are identified as “source,” and then their connectivity with other neighboring cell types is depicted.

A number of groups model spatial patterns of gene expression as derived from predefined processes. For example, spatialDE uses a random effect model that contains two terms, corresponding to the spatial and nonspatial component, respectively (Svensson et al. 2018a). The spatial component can be specified as various forms such as linear, periodic, or a Gaussian process. The degree of spatial variability is then quantified by the ratio of the variance explained by these two terms. SOMDE uses a similar approach but increases computational efficiency by first compressing spatial information by using a self-organizing map-based transformation (Hao et al. 2021b). Trendsceek models spatial patterns as a marked point process (Edsgärd et al. 2018). SPARK models spatial count data through generalized linear spatial models with an additional step to calibrate *P*-value calculation (Sun et al. 2020). Some methods are mainly concerned about local continuity. As an example, binSpect detects spatially coherent genes as those that tend to be coexpressed in neighboring cells, using a spatial network formulation (Dries et al. 2021). Yet another approach is to quantify spatial structure in terms of diffusive steps it takes to reach a homogeneous configuration (Anderson and Lundeberg 2021). The identification of spatially coherent genes can in turn inform cell-state spatial pattern detection (Zhu et al. 2018). Alternatively, the spatial gene and domain detection steps are inferred simultaneously (Hu et al. 2020a). As a concrete example, binSpect was used to identify genes with a spatial coherent pattern in the MERFISH coronal brain slice data, and top-ranked genes are shown in Figure 5F.

## Subcellular structure analysis

**Figure 6.**
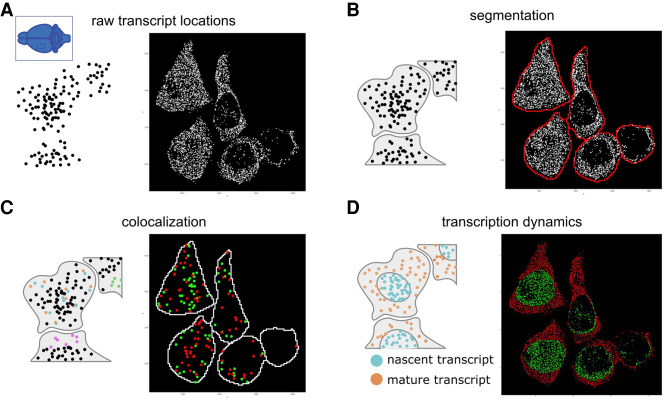
Schematic diagram for spatial transcriptomics analysis at subcellular resolution. (*A*) For spatial data at subcellular resolution, each dot typically represents a single transcript or, alternatively, a spatial unit that is well below the cell size. (*B*) The location of each transcript, along with its gene identity, can be used as input to try and segment each cell. (*C*) Individual transcripts can be colocalized with other transcripts (orange and blue) or with itself (green) or can be found at specific subcellular structures (pink at membrane). (*D*) Transcription dynamics from individual or multiple genes can be inferred from the location of transcripts. Here nascent transcripts are typically found in the nucleus (blue), whereas processed transcripts are found in the cytoplasm (orange). The ratio between the two can provide an estimate for the RNA velocity. Examples for each analysis are provided on the *right* of each panel using the seqFISH+ data set from the mouse somatosensory cortex.

With the advancement of newer technologies, it is now possible to study subcellular transcript organizations. In addition to FISH-based methods (Lubeck et al. 2014; Chen et al. 2015; Shah et al. 2016; Codeluppi et al. 2018; Moffitt et al. 2018; Eng et al. 2019; Kishi et al. 2019; Goh et al. 2020), which are well known to have single-molecule resolution, ISS approaches (Lee et al. 2014; Wang et al. 2018; Qian et al. 2020; Hu et al. 2020b; Alon et al. 2021; Fu et al. 2021) also offer very high resolution. In addition, high-density array or bead-based technologies (Vickovic et al. 2019; Alon et al. 2021; Chen et al. 2021; Stickels et al. 2021) have also enabled subcellular resolution. Here we use the seqFISH+ mouse somatosensory cortex data set to illustrate some key concepts of subcellular data analysis (Fig. 6). In a data set with subcellular resolution, each point typically represents a single transcript (Fig. 6A). Analyzing the subcellular gene expression patterns can be used as an alternative approach for spatial analysis but also can be used to enhance the accuracy of cell segmentation (Fig. 6B). Finally, subcellular localization of RNA transcripts can also be used to gain biological insights that are not possible through cell-level analyses. Individual spatial relationships between genes or between genes and subcellular structures are found through analysis of colocalization patterns (Fig. 6C) and transcription dynamics within each cell (Fig. 6D).

A number of methods have been developed to use subcellular gene expression patterns to circumvent cell segmentation, which can be challenging. For example, SSAM assigns cell type labels directly to pixels without cell segmentation (Park et al. 2021). stLearn uses a similar approach but further clusters spatially proximal pixels that are assigned to the same cell type (Pham et al. 2020). Spage2vec also uses a similar approach but adapts a neural network formulation (Partel and Wählby 2021). Alternatively, supervised cell type mapping strategies based on known cell type–specific signatures have been developed. For example, a naive Bayes model is used to assign cell types for HDST data (Vickovic et al. 2019). Subcellular gene expression patterns can in turn be used to improve cell segmentation. For example, Baysor models the subcellular gene expression patterns by using a Markov random field model and further integrates cell shape labeling information (such as DAPI) to improve cell segmentation accuracy (Petukhov et al. 2020). Sparcle (Prabhakaran et al. 2021) uses a Dirichlet process mixture model instead as well as the transcripts’ distance between neighboring cells and adjacent transcripts to enhance cell segmentation. JTSA uses an EM algorithm to iteratively improve pixel-level gene expression profile classification and cell-boundary annotations (Littman et al. 2021).

Analysis of the subcellular patterns of gene expression can also provide new biological insights. For example, an in situ RNA velocity approach has been developed to use subcellular RNA localization information to infer the transcription rates (Xia et al. 2019). Because newly transcribed RNAs are cumulated in the nucleus, whereas mature mRNA needs to be transported to the cytoplasm for translation (Fig. 6D), the relative composition of nuclear versus cytoplasmic transcripts associated with each gene can be used to estimate the transcriptional activity. This is performed by using a similar mathematical formulation as in the original RNA velocity paper (La Manno et al. 2018).

In addition, colocalized mRNA species in the cytoplasm can be identified with high resolution by using direct proximity labeling of RNA using the peroxidase enzyme APEX2, a method called APEX-seq (Fazal et al. 2019). Analysis of the resulting data identifies a remarkable correspondence between colocalized RNA with known protein colocalization patterns (Fazal et al. 2019), suggesting RNA colocalization may facilitate local protein translation and complex formation (Fig. 6C). Also, mRNAs enriched in nuclear locations tend to code for proteins enriched in nuclear speckles and nucleoplasm. Alternatively, subcellular RNA colocalization can also be detected by ATLAS-seq, which uses sucrose density gradient ultracentrifugation followed by RNA sequencing (Adekunle and Wang 2020). In this study, it was also found that RNAs tended to colocalize with other RNAs in similar protein complexes, in cellular compartments, or with similar biological functions.

## Understanding how cells communicate with the tissue environment

**Figure 7.**
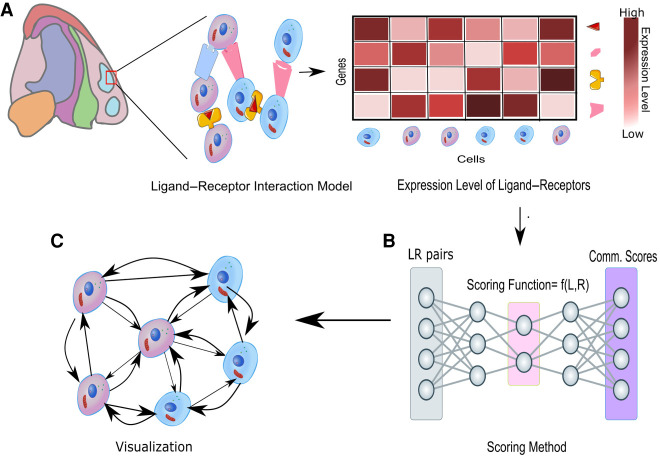
Cellular communication inferred from ligand–receptor interactions. The known ligand–receptor interaction pairs are first explored using their gene expression profiles and then passed to a computational tool to generate communication scores that explain connectivity between and within each cell type as shown in *A*. A spatial graph can be constructed with these scores between different cell types as shown in *B* and *C*.

An important goal of ST analysis is to study how cells communicate with the tissue environment (Fig. 7). Cellular behavior can be significantly affected by the tissue environment through direct physical interactions, secreted molecules, or interactions with the extracellular matrix (Fig. 7A). For example, the development of tumor vasculature can significantly promote tumor growth, whereas enriched immune cells in tumor microenvironments could significantly control its proliferation. Cell–cell communications are often spatially coordinated and can be highly cell type–specific (Armingol et al. 2021). Thus, the variation of cell type compositions could lead to significant changes of gene expression even within the same cell type (Fig. 7B,C).

Giotto introduces a two-way comparison method to identify interaction changed genes by comparing the gene expression pattern between subsets of cells within the same cell type but surrounded by different neighboring cells (Dries et al. 2021). Of note, using the spatial information can significantly reduce the number of false-positive ligand–receptor activity predictions compared with using gene expression information alone. A similar approach is used in CellPhoneDB v3.0 (Garcia-Alonso et al. 2021). In this study, the ST data do not have single-cell resolution. To overcome this challenge, the investigators applied Cell2location (Kleshchevnikov et al. 2020) to infer the location of different cell types before comparing gene expression patterns associated with different cell neighborhoods. Alternative approaches have been used to quantify the effect of neighboring cell types, including convolutional neural networks (Li et al. 2020; Yuan and Bar-Joseph 2020), optimal transport (Cang and Nie 2020), and multioutput regression (Li et al. 2021). Another approach is to explicitly decompose a gene expression profile into spatial and nonspatial components and then use the cell type composition in the neighborhood to estimate the spatial components (Arnol et al. 2019). The analysis of ligand–receptor interactions has also been extended to include the effect of cofactors in the multiunit protein complexes to enhance prediction accuracy (Jin et al. 2021). Of note, algorithms have also been developed to reconstruct spatial locations from cell–cell interaction patterns (Ren et al. 2020).

## Integrative exploratory tools for spatial data analysis and visualization

**Figure 8.**
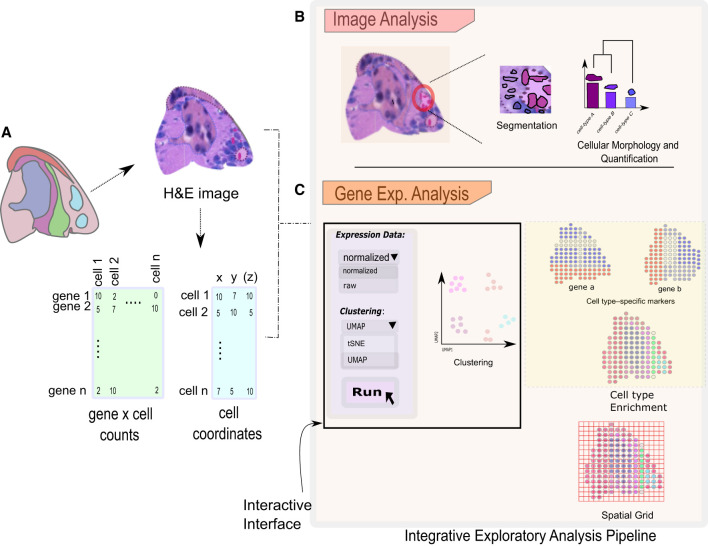
An overview of interactive exploratory analysis pipeline. The integrative and interactive pipeline with several options can be used to analyze the spatial data sets. (*A*) Spatial data analysis starts with importing and processing raw data sets. The analysis can then be subdivided into image-based analysis (*B*) and gene expression–based analysis (*C*). Analysis based on images such as cell segmentation and morphological quantification is available to investigate the cellular intricacies in a selected section of a tissue. Gene expression–based analysis consists of several approaches such as clustering, spatial network construction, and cell type enrichment to visualize gene expression patterns. An interactive graphical interface makes these methods easier accessible for novice users.

To effectively use and disseminate new methods that are being developed to achieve a specific spatial data analysis task, it becomes increasingly important to develop the necessary data structures and tools to work with them at a larger scale. Biologists will benefit from having integrative and interactive pipelines that allow them to conduct various analysis steps, from importing raw data (Fig. 8A) to image analysis (Fig. 8B), followed by the production of final analysis results and figures ready for publication (Fig. 8C), ideally on their personal computer. Method developers can build on previous spatial structures or make their new methods easily available to a larger audience. Currently, there are a number of comprehensive toolboxes available, as described below. Here we will not discuss the specific steps necessary to process raw data, such as images or sequence reads, because they are typically specific for each technology, but we limit the survey to tools that are designed for downstream exploratory data analysis. Most of the code for these tools is written in the popular programming languages R (R Core Team 2020) or Python or with a combination of both by making use of recently developed interfaces such as reticulate (https://github.com/rstudio/reticulate) or basilisk (http://basilisk.fr), which allow developers to fully benefit from the strengths of both worlds.

Giotto (Dries et al. 2021) is an R package that implements this latter strategy and has been shown to work on a large variety of ST technologies. It can also be applied to antibody-based protein multiplexed imaging technologies, although the latter is beyond the scope of this review. At its core, Giotto consists of an object specifically designed for spatial data. At minimum, this object stores both the count matrix and the accompanying 2D or 3D coordinates of the spatial units, either individual cells or spatial aggregates as explained earlier. It provides routine analyses such as filtering, clustering, and cell type annotation and presents spatial relationships as a network graph or through a spatial grid. This network forms the starting point for many new specific spatial analyses and facilitates the integration of other established algorithms through the creation of simple wrappers. For visualization purposes, raw images of the profiled tissue can be stored and used to overlay the obtained spatial results. In parallel, Giotto offers a browser-based visualization tool, Giotto Viewer, that allows users to export their obtained results and explore the spatial data set in an interactive manner.

Seurat is better known as a popular R package for scRNA-seq analysis, but it commenced to offer some advanced functionalities through its spatial branch (Hao et al. 2021a). The functions are specific to spatial data visualization and the identification of spatial expression patterns through the usage of established methods. Furthermore, other tools such as STUtility (Bergenstråhle et al. 2020) and SPATA (Kueckelhaus et al. 2020a) have built on top of the rich and performant data structure of Seurat to create more comprehensive pipelines that are currently specific for the ST technology. STUtility (Bergenstråhle et al. 2020) was developed specifically for the ST technology and offers a wide variety of imaging and data analysis methods that are targeted for this approach. Similarly, SPATA (Kueckelhaus et al. 2020) focuses on ST data and was developed to facilitate integration with the popular R packages Seurat and Monocle. Besides visualization and common data analysis functions, SPATA also has a rich repertoire of interactive methods to identify or delineate spatial trajectories.

Squidpy (Palla et al. 2021) is the spatial counterpart of SCANPY (Wolf et al. 2018), the popular Python library for scRNA-seq analysis, and was created by the same laboratory. Similar to Giotto, it starts by representing the spatial information through a spatial network and offers a large variety of downstream spatial analysis. In contrast to other toolboxes, it also provides analysis at the image level, which ranges from typical tasks such as segmentation or registration to more advanced ways of extracting and using morphology information in downstream analysis. Stlearn (Pham et al. 2020) is another Python library for ST data analysis with a specific focus on integrating both gene expression and image information through a joint representation.

Most of these packages or toolboxes are developed in independent laboratories, which results in multiple different data structures that do not necessarily share the same data format. To overcome some of these challenges, the R/Bioconductor community is engaged in the careful design of generally applicable data structures and has recently published the first version of the spatialExperiment class (Righelli et al. 2021). This is a new S4 class that extends the popular singleCellExperiment class (Amezquita et al. 2020) and is designed to operate with several types of ST data sets, including at both multi- and subcellular resolution. Several spatial R packages already exist that use this data structure, such as SpatialLIBD (Pardo et al. 2021) and Spaniel (Queen et al. 2019), which both excel in the creation of interactive R/Shiny apps to visualize ST data sets. All together, these efforts could contribute to the promotion of interoperability between these different toolboxes in the future.

## Discussion

The rapid development of ST technologies has provided new opportunities and challenges for data analysis. As summarized above, there has been a lot of progress in this domain in recent years. Novel methods have been developed for attacking various ST-specific challenges. Integrative software packages have enabled biologists to easily analyze their own data from beginning to the end and to interactively explore the data via interactive visualization. Together, these tools play important roles for making the ST technologies broadly applicable.

Since the pioneering work by Ramón y Cajal, it has been standard practice to classify different cell types based on morphological changes. In recent years, there has been a paradigm shift by classifying cell types based on transcriptomic profiles, sometimes complemented by additional molecular modalities. Owing to the rapid development of ST technologies, it is now possible to perform both transcriptomic profiling and morphology analyses for the same cells, thereby providing a great opportunity to systematically investigate the relationship between these two fundamentally different approaches. A few methods have recently been developed that focus on integration of both modalities (He et al. 2020; Tan et al. 2020; Gerbin et al. 2021). Although not directly related to spatial transcriptomics, an interesting finding from living imaging analysis indicates that changes in morphology might even predict cell fate or state before this can be observed in the transcriptomic output (Buggenthin et al. 2017). Future work, including the reconstruction of complete 3D tissues using CODA (Kiemen et al. 2020), in this direction will help in bridging the gap between communities.

An exciting new direction that is not covered here is spatial multiomics. New technology development has made it possible to profile multiple modality information in the same cells while preserving information, such as protein and RNA (Saka et al. 2019; Liu et al. 2020; Merritt et al. 2020; Takei et al. 2021), intron and mature mRNA (Shah et al. 2018; Mateo et al. 2019; Su et al. 2020), DNA, and RNA (Mondal et al. 2018; Mateo et al. 2019; Su et al. 2020; Takei et al. 2021). These technologies have made it possible to analyze the correlation between different molecular modalities and offer mechanistic insights. Analyzing such data requires development of novel computational methods and toolboxes. In fact, a number of multiomic analysis methods have already been developed for sequencing-based assays (Argelaguet et al. 2018; Butler et al. 2018; Haghverdi et al. 2018; Barkas et al. 2019; Hie et al. 2019; Korsunsky et al. 2019; Stuart et al. 2019; Welch et al. 2019; Biancalani et al. 2020; Peng et al. 2021). The readers are referred to published reviews to learn more about this topic (Stuart and Satija 2019; Ma et al. 2020; Forcato et al. 2021). However, further development is needed to incorporate the spatial context.

In sum, spatial technologies have brought many new challenges and opportunities. We believe that computational method development will continue to play a critical role in translating the promise of spatial technologies to reality by providing important tools for the analysis, visualization, and interpretation of new data.
